# Age-related prognoses in a Luxembourgish breast cancer cohort

**DOI:** 10.3389/fonc.2026.1763412

**Published:** 2026-06-22

**Authors:** Allini Mafra, Sophie Couffignal, René Aloisio da Costa Vieira, Caroline Duhem, Claudine Backes

**Affiliations:** 1 Registre National du Cancer du Luxembourg (RNC), Strassen, Luxembourg; 2 Public Health Expertise Unit (PHE), Luxembourg Institute of Health (LIH), Strassen, Luxembourg; 3 Cancer Epidemiology and Prevention Group (EPICAN), Department of Precision Health (DoPH), Luxembourg Institute of Health (LIH), Strassen, Luxembourg; 4 Postgraduate Program in Tocogynecology, Botucatu School of Medicine, Botucatu, SP, Brazil; 5 Postgraduate Program in Oncology, Barretos Cancer Hospital, Barretos, SP, Brazil; 6 Division of Breast Surgical Oncology, Department of Surgical Oncology, Muriaé Cancer Hospital, Muriaé, MG, Brazil; 7 Centre Hospitalier du Luxembourg, CHL Kriibszentrum, Department of Hemato-Oncology, Luxembourg City, Luxembourg

**Keywords:** age-related prognoses, breast neoplasms, screening programs, survival outcomes, tumour biology

## Abstract

**Background:**

Breast cancer (BC) is the most common cancer among women worldwide, and age significantly influences prognosis. In July 2024, Luxembourg expanded BC screening programme (Programme Mammographie, PM), changing the eligible age from 50–69 to 45–74 years. This study assessed age-related BC prognosis before expansion.

**Methods:**

This retrospective cohort study analysed 3,003 women diagnosed with invasive BC in Luxembourg (2013–2018) using National Cancer Registry data. Patients were stratified into four age groups: <40, 40–49, 50–69, and ≥70 years. Tumour characteristics, treatment, and five-year overall survival (OS) were compared across groups using Kaplan-Meier and Cox proportional hazards models.

**Results:**

Younger patients (<40 years) showed higher triple-negative tumours (24%, P<0.001) and chemotherapy use (78%). Five-year OS varied by age group, with the highest rates in women aged 40–49 (95.3%) and 50–69 (92.3%), followed by those <40 (91.0%), and lower survival in women ≥70 (65.4%) (P<0.001), a finding that partly reflects competing causes of mortality in this age group rather than BC prognosis alone. Screen-detected cases had better survival (95.8%). Advanced stage (hazard ratio [HR]=2.92, 95% confidence interval [CI]: 2.05–4.17) and triple-negative subtype (HR = 2.39, 95% CI: 1.69-3.38) linked to worse prognosis. Mastectomy (HR = 2.04) and absence of surgery (HR = 8.58) were associated with poorer survival, likely reflecting disease complexity rather than causal treatment effects.

**Conclusion:**

Age at diagnosis influences BC prognosis through distinct tumour characteristics, including molecular subtype, histology, clinical stage, and mode of detection, which collectively impact treatment and survival. Women aged 40–49 and 50–69 achieved the most favourable survival outcomes. Among women in the established screening target group (50–69 years), early detection through organised mammography was associated with particularly high survival, reinforcing the value of standardised early detection in this group. Younger patients presented with more aggressive tumour biology, while older patients showed lower OS, an estimate that substantially reflects competing causes of death, including cardiovascular disease and other malignancies, in addition to any BC-attributable mortality disadvantage. These findings establish a baseline for evaluating expanded PM and highlight the need for age-specific strategies.

## Introduction

Breast cancer (BC) is a major global health challenge and the most common cancer among women worldwide. In 2022, within the European Union (EU-27), an estimated 380,000 new BC cases and 97,000 related deaths were reported, according to the European Cancer Information System (ECIS) data (1). Global projections estimate an increased incidence and mortality by 2045 (2, 3). In Luxembourg, BC is the most frequent cancer type in women, representing 17% of all new cancer cases in females, and remains the leading cause of cancer mortality in women, despite significant decreasing mortality trends between 1998–2021 (4–6). BC remains an important public health challenge with a multifactorial aetiology involving genetic, hormonal, and environmental influences. Established risk factors include early menarche, late menopause, nulliparity, hormone replacement therapy, alcohol consumption, and obesity (7, 8).

Age is a well-documented risk factor that influences BC incidence and prognosis. Half of the BC cases in women occurred in the 45–69 age range (9–11). This includes women aged 45–49, recently incorporated into Luxembourg’s national screening program (in French: *Programme Mammographie*, PM), and those aged 50–69, the core target of European recommendations for mammographic screening. Despite the European Council’s efforts since 2003, the implementation of organised mammography screening varies across Europe, depending mainly on policies, healthcare organisations, and resources. Since the 2006 European Guidelines on Breast Cancer Screening and Diagnosis (12), new evidence on BC and innovation has been analysed by the European Commission Initiative on Breast Cancer (ECIBC), which has developed new evidence-based recommendations. These recommendations support expanding the organised screening programmes from women aged 50–69 to 40–74 years for average-risk cases, while addressing digital mammography screening, other methodologies, and screening frequency.

Routine screening is generally not recommended for women under 40 or over 70 years of age. It may be considered based on an individualised risk assessment, incorporating factors such as life expectancy, comorbidities, and functional status (13, 14).

Studies suggest that BC diagnosed at a younger age is more aggressive, with a higher prevalence of triple-negative tumours (15–17). Survival disparities in older patients remain debated, as comorbidities and treatment decisions impact outcomes (18–22). Despite research on age-related BC prognosis, findings remain inconsistent due to variations in cohort selection, age categories, and adjustments for tumour biology and treatment.

Luxembourg has one of Europe’s most dynamic populations, which has increased by 84.3% since 1981 (23), nearly 15 times the European Union’s growth. The average age of the population rose to 39.7 years in 2023, from 38.7 years in 2011 (23). These demographic shifts highlight the need for age-adapted cancer prevention strategies. Key determinants of BC survival include both organised mammography screening and access to effective treatment (24). European screening typically targets women aged 50–69, although support is growing for including women aged 40–49, 45–49, and 70–74 years (25, 26). Globally, the organisation and coverage of BC screening programmes vary considerably. In the United States, professional guidelines recommend annual or biennial mammography for women aged 40–74, with individual risk-based decisions endorsed by major clinical societies (27). Australia and Canada implement organised biennial screening programmes primarily targeting women aged 50–74. In contrast, many low- and middle-income countries (LMICs) lack population-based organised mammography due to limited resources and infrastructure, relying instead on clinical breast examination or opportunistic imaging (9, 28–30). Within Europe, the systematic monitoring and evaluation of organised screening programmes are recommended as an integral component of quality assurance, in line with the European Guidelines for Quality Assurance in Breast Cancer Screening and Diagnosis (31) and performance indicator frameworks developed for EU Member States (32). These global disparities, together with the European commitment to programme evaluation, underscore the importance of assessing age-related outcomes within national programmes such as Luxembourg’s PM, where high-quality registry data enable rigorous analysis.

Luxembourg recently (July 2024) expanded its PM to women aged 45–74 years, lowering the minimum age from 50 to 45 years and raising the upper limit from 69 to 74 years (33). Including younger women (45–49 years) aims to enhance early detection for improved survival rates. Extending screening to ages 70–74 recognises older women’s potential benefit from early-stage diagnosis. However, evidence on the long-term benefits remains limited, requiring an assessment of whether expanded screening improves outcomes or increases overdiagnosis (33, 34). The PM has achieved moderate but improving participation rate, higher than those commonly observed in neighbouring countries, yet still below the European recommendations of 70% for an acceptable rate and 75% for a desirable participation rate (35). The participation rate reported for the organised PM does not include opportunistic screening, for which the absence of a specific classification code prevents its precise quantification. Estimates suggest that overall screening coverage of the target population (organised and opportunistic combined) is approximately 70%, close to the acceptable European threshold (35). Although difficult to quantify precisely, opportunistic screening through medical prescriptions in outpatient settings likely contributes to early BC detection in Luxembourg. National guidance from 2018 supports personalised screening based on risk profiles, including family history, breast density, and genetic predisposition (36).

This policy change underscores the importance of establishing a baseline of age-related prognosis before the expansion. Luxembourg’s unique demographic profile, marked by rapid growth, high immigration, and population ageing, reinforces the need to understand survival differences across age groups. Therefore, this study aimed to evaluate the impact of age at diagnosis on BC prognosis in Luxembourg between 2013 and 2018, using high-resolution data from the National Cancer Registry (in French: *Registre National du Cancer*, RNC).

## Methods

This retrospective cohort study included female patients diagnosed or treated in Luxembourg with pathologically confirmed invasive BC (ICD-O-3/3 (37)) between 2013 and 2018. The PM primarily targeted women aged 50–69. Cases with sarcomas/lymphomas, second primary BC, or lost to follow-up at baseline were excluded (**Supplementary Figure 1**). Patients were stratified into four age groups based on diagnosis age: <40, 40–49, 50–69, and ≥70 years.

Luxembourg’s National Cancer Registry (In French: Registre National du Cancer, RNC) is the official data collection of all new cancer cases diagnosed and/or treated in Luxembourg for both residents and non-residents, in order to have an overview of all cancer patients being cared for in Luxembourg.

The RNC was established following the Grand-Ducal Regulation of 18 April 2013, making it one of the youngest cancer registries in Europe. As a population-based cancer registry (PBCR) it operates as multi-source system drawing on hospital-based data, data from the National Health Fund (in French: *Caisse Nationale de Santé*, CNS) and the National Health Insurance Medical Inspection Service (in French: *Contrôle Médical de la Sécurité Sociale*, CMSS), and the National Registry of Causes of Death of the Luxembourgish Ministry of Health (in French: *Registre national des causes de décès*). Data codification follows international classifications, including the ICD-O-3 and the UICC (Union for International Cancer Control) TNM (Tumour, Node, Metastasis) Classification of Malignant Tumours, enabling international comparisons (37, 38). Recording and validation rules follow recommendations from the European Network of Cancer Registries (ENCR) and the International Association of Cancer Registries (IACR). Data quality indicators, including morphological verification rates, death-certificate-only rates, and mortality-to-incidence ratios, are documented in national epidemiological publications. For breast cancer specifically, the morphological verification rate was 95.5% and the death-certificate-only rate was 3.8% over the period 2014–2021, both within internationally acceptable ranges (6, 35, 39).

Detection mode was defined for all BC patients 50–69 years at the moment of the mammography based on linkage between RNC data and PM participation records (35, 40). For women who participated in the PM within 50–69 age range and were subsequently diagnosed at ≥70, age classification was based on diagnosis age, while detection mode was assigned according to their most recent PM participation record. Screen-detected cases were those with a mammography performed within the PM within two years before diagnosis at which an abnormality was detected. Interval-detected cases were operationally defined as those with PM mammography within two years prior to diagnosis, with no recorded abnormality (noting that this definition may include a proportion of true interval cancers as well as cases where the preceding examination was performed outside the organised programme). Diagnosis-detected cases were those without a recorded mammography within two years prior to diagnosis.

Tumours were classified into five molecular subtypes based on hormone receptor status, HER2 expression, and Ki67 levels (41). “Luminal A” if they were oestrogen receptor (ER)- and/or progesterone receptor (PR)-positive, human epidermal growth factor receptor 2 (HER2)-negative, with Ki-67 proliferation index <14%. “Luminal B HER2-negative” tumours were ER/PR-positive, HER2-negative, with Ki-67 index ≥14%. “Luminal B HER2-positive” tumours were ER/PR positive and HER2 overexpression. “Triple-negative tumours” were negative for ER, PR, and HER2, while “HER2-positive (non-luminal)” tumours were HER2-positive with ER/PR negative. Ki-67 data were used to support subtype classification (≥14% threshold for the Luminal B HER2-negative vs. Luminal A distinction, per St Gallen 2011 criteria (41)). This five-category classification follows research-oriented definitions (41), but may not fully reflect clinical practice in Luxembourg, where Luminal B HER2-positive tumours are not consistently reported as a separate group in routine pathology reports.

Clinical stage followed the TNM classification system (38). Metastatic sites were categorised as visceral (liver and/or lungs); and non-visceral (brain, bone, and/or lymphatic system). For multiple metastases, visceral was selected for categorisation. BC treatment is individualised based on clinical stage, breast/tumour ratio, lymph node status, tumour characteristics, age, comorbidities, and access to multimodal therapy (42). Treatment modalities included surgery (breast-conserving, mastectomy, or none), radiotherapy, chemotherapy, hormonal therapy, all recorded as binary variables (yes/no).

Patient demographics and clinical characteristics were compared across age groups using the chi-squared test, with Fisher’s exact test for cells with expected frequency <5. Follow-up time was defined as the interval from the date of diagnosis to death from any cause or the last follow-up. Patients lost to follow-up were censored on the last known contact. For survival analysis, patients alive with fewer than 60 months of follow-up at data extraction were administratively censored at their last recorded contact. Patients who had died or were alive with ≥60 months of follow-up were considered to have completed follow-up. A sensitivity analysis compared these group (**Supplementary Table 1**).

Overall survival (OS) was estimated using Kaplan-Meier curves, with differences assessed using log-rank tests. Cox proportional hazards models were used to estimate the hazard ratios (HRs) and 95% confidence intervals (CIs). Statistical analyses were performed using R software (version 4.4.2; R Foundation for Statistical Computing, Vienna, Austria). All P-values were two-sided, with significance set at <0.05. Missing values were handled using complete-case analysis in all Cox proportional hazards models; patients with missing values for any covariate included in a given model were excluded from that analysis. Variables with low completeness, specifically BRCA1 and BRCA2 mutation status, were not included in multivariate models, as genetic testing is performed selectively based on clinical indication rather than routinely. The potential impact of missing data on model estimates is acknowledged as a limitation.

The study was conducted in accordance with ethical guidelines and approved by the National Research Ethics Committee of Luxembourg (in French: *Comité National d’Éthique de Recherche* (CNER); protocol number: 202212/01; approval date: December 2022) and the RNC Scientific Steering Committee.

## Results

**Figure 1:**
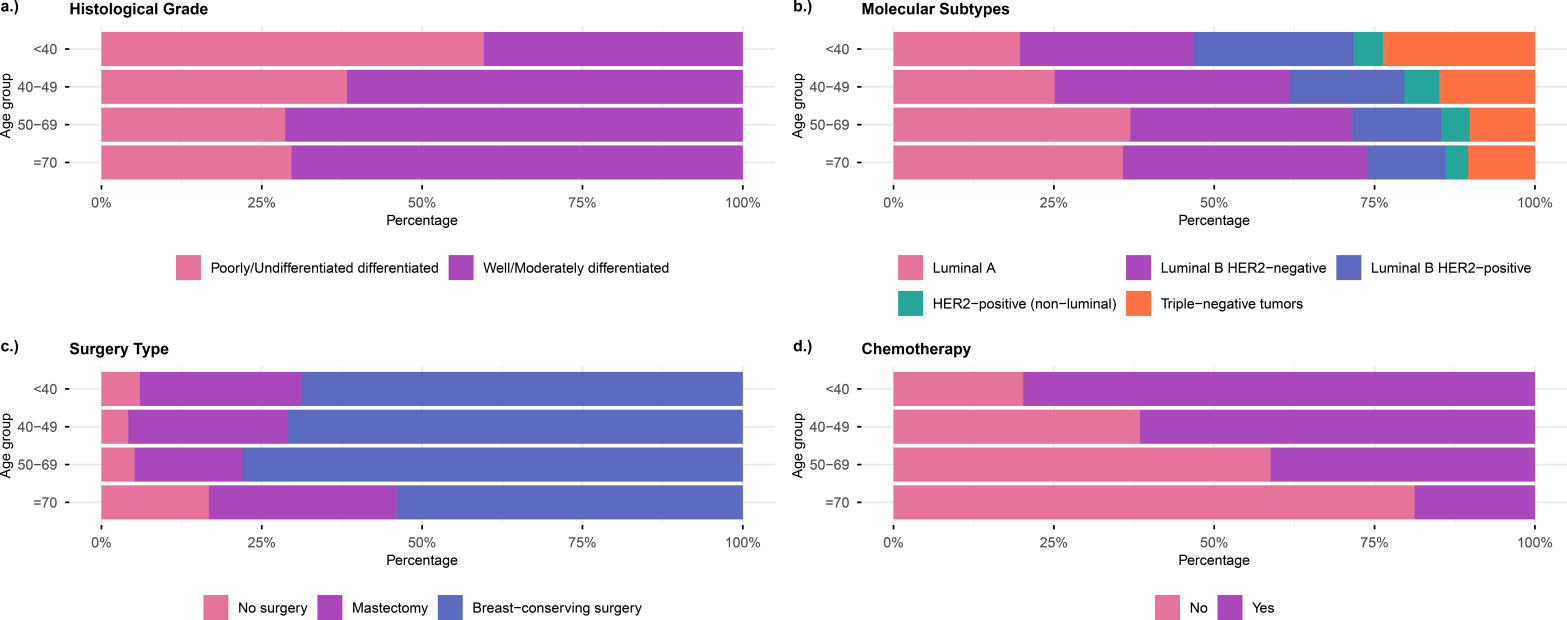
Distribution of **(A)** Histological grade, **(B)** Molecular subtypes, **(C)** Surgery type and **(D)** Chemotherapy by age groups.

A total of 3,003 patients with invasive BC were analysed, with a median follow-up of 68 months (interquartile range (IQR): 51–81). As shown in **Table 1**, 6% of patients were diagnosed before age 40, while 47% were in the 50–69‐year age range. **Table 1**; **Figure 1** show that younger patients (<40 years) were significantly more likely to present with high‐grade tumours, with a higher proportion of poorly differentiated or undifferentiated histology (P<0.001). The molecular subtype varied with age. Triple‐negative tumours were most frequent in the <40 group (24%), whereas luminal A subtypes predominated in the 50–69 groups (37%). In the ≥70 group, Luminal B HER2-negative was the most frequent subtype (38%), slightly exceeding Luminal A (36%), with the predominance of luminal subtypes collectively consistent across both older age groups (P<0.001). Treatment patterns differed; chemotherapy use was higher in patients <40 years (80%) than in those ≥70 years (19%), and mastectomy rates were highest in women ≥70 years (29%) and lowest in women aged 50-69 (17%). A supplementary analysis of chemotherapy intent (**Supplementary Table 8**) identified 258 patients (8.6%) who received neoadjuvant chemotherapy, with the highest proportion in women aged <40 years (22.6%), declining progressively with age (≥70: 2.9%).

**Table 1:** Demographic and clinical characteristics of BC cases stratified by age at diagnosis.

Variable / Categories	All ages N = 3,003^1^	<40 N = 186^1^	40–49 N = 608^1^	50–69 N = 1,419^1^	≥70 N = 790^1^	p-value^2^
**Country of Residence at Diagnosis**						**<0.001**
Luxembourg	2,584 (86%)	144 (77%)	491 (81%)	1,229 (87%)	720 (91%)	
Other country	419 (14%)	42 (23%)	117 (19%)	190 (13%)	70 (8.9%)	
**Year of diagnosis**						0.4
2013-14	984 (33%)	56 (30%)	213 (35%)	461 (32%)	254 (32%)	
2015-16	973 (32%)	71 (38%)	183 (30%)	452 (32%)	267 (34%)	
2017-18	1,046 (35%)	59 (32%)	212 (35%)	506 (36%)	269 (34%)	
**Detection mode**						NA
Screen-detected	598 (39%)	NA	NA	589 (42%)	9 (9.1%)	
Interval-detected	214 (14%)	NA	NA	206 (15%)	8 (8.1%)	
Diagnosis-detected	704 (46%)	NA	NA	622 (44%)	82 (83%)	
Missing values	1,487	NA	NA	2	691	
**Histological diagnosis**						**<0.001**
Ductal carcinoma	1,710 (57%)	122 (66%)	342 (56%)	801 (56%)	445 (56%)	
Lobular carcinoma	1,119 (37%)	51 (27%)	240 (39%)	567 (40%)	261 (33%)	
Others	174 (5.8%)	13 (7.0%)	26 (4.3%)	51 (3.6%)	84 (11%)	
**Differentiation grade**						**<0.001**
Well/Moderately differentiated	1,607 (67%)	61 (40%)	313 (62%)	822 (71%)	411 (70%)	
Poorly/Undifferentiated differentiated	787 (33%)	90 (60%)	194 (38%)	330 (29%)	173 (30%)	
Missing values	609	35	101	267	206	
**SBR**						**<0.001**
Grade I	347 (18%)	7 (6.3%)	70 (17%)	191 (20%)	79 (17%)	
Grade II	1,040 (54%)	42 (38%)	194 (48%)	539 (57%)	265 (57%)	
Grade III	537 (28%)	62 (56%)	137 (34%)	219 (23%)	119 (26%)	
Missing values	1,079	75	207	470	327	
**Multifocality**						**<0.001**
Presence	1,046 (35%)	79 (42%)	247 (41%)	497 (35%)	223 (28%)	
Absence	1,957 (65%)	107 (58%)	361 (59%)	922 (65%)	567 (72%)	
**Laterality**						0.8
Right	1,495 (50%)	87 (47%)	303 (50%)	716 (51%)	389 (49%)	
Left	1,503 (50%)	99 (53%)	305 (50%)	701 (49%)	398 (51%)	
Missing values	5	0	0	2	3	
**Clinical T**						**<0.001**
T1	1,582 (58%)	78 (48%)	319 (59%)	830 (65%)	355 (49%)	
T2	856 (32%)	70 (43%)	185 (34%)	341 (27%)	260 (36%)	
T3	121 (4.5%)	12 (7.3%)	29 (5.3%)	49 (3.8%)	31 (4.3%)	
T4	150 (5.5%)	4 (2.4%)	11 (2.0%)	60 (4.7%)	75 (10%)	
Missing values	294	22	64	139	69	
**Clinical N**						
N0	2,232 (77%)	118 (66%)	446 (75%)	1,117 (81%)	551 (74%)	
N1	588 (20%)	56 (31%)	139 (23%)	221 (16%)	172 (23%)	
N2	28 (1.0%)	2 (1.1%)	4 (0.7%)	11 (0.8%)	11 (1.5%)	
N3	44 (1.5%)	2 (1.1%)	6 (1.0%)	22 (1.6%)	14 (1.9%)	
Missing values	111	8	13	48	42	
**Clinical M**						**<0.001**
M0	2,803 (94%)	175 (96%)	586 (97%)	1,335 (95%)	707 (91%)	
M1	168 (5.7%)	7 (3.8%)	18 (3.0%)	69 (4.9%)	74 (9.5%)	
Missing values	32	4	4	15	9	
**Site of metastasis**						0.15
Visceral (liver and/or lung)	37 (22%)	0 (0%)	5 (28%)	11 (16%)	21 (29%)	
Non-visceral (brain, bone and/or lymphatic system)	129 (78%)	7 (100%)	13 (72%)	57 (84%)	52 (71%)	
NA or Missing values	2,837	179	590	1,351	717	
**Clinical stage**						**<0.001**
I	1,415 (52%)	63 (38%)	280 (51%)	757 (59%)	315 (43%)	
II	979 (36%)	84 (51%)	218 (40%)	398 (31%)	279 (38%)	
III	172 (6.3%)	12 (7.2%)	29 (5.3%)	64 (5.0%)	67 (9.1%)	
IV	168 (6.1%)	7 (4.2%)	18 (3.3%)	69 (5.4%)	74 (10%)	
Missing values	269	20	63	131	55	
**Molecular subtypes**						**<0.001**
Luminal A	745 (33%)	30 (20%)	116 (25%)	393 (37%)	206 (36%)	
Luminal B HER2-negative	797 (35%)	41 (27%)	169 (37%)	368 (35%)	219 (38%)	
Luminal B HER2-positive	340 (15%)	38 (25%)	83 (18%)	148 (14%)	71 (12%)	
HER2-positive (non-luminal)	99 (4.4%)	7 (4.6%)	25 (5.4%)	47 (4.4%)	20 (3.5%)	
Triple-negative tumours	273 (12%)	36 (24%)	69 (15%)	108 (10%)	60 (10%)	
Missing values	749	34	146	355	214	
**BRCA1**						0.5
Negative	189 (81%)	41 (76%)	75 (83%)	67 (81%)	6 (100%)	
Positive	44 (19%)	13 (24%)	15 (17%)	16 (19%)	0 (0%)	
Missing values	2,770	132	518	1,336	784	
**BRCA2**						0.3
Negative	197 (85%)	41 (79%)	78 (90%)	72 (84%)	6 (86%)	
Positive	35 (15%)	11 (21%)	9 (10%)	14 (16%)	1 (14%)	
Missing values	2,771	134	521	1,333	783	
**Surgery**						**<0.001**
Breast-conserving surgery	2,042 (70%)	126 (69%)	427 (71%)	1,089 (78%)	400 (54%)	
Mastectomy	647 (22%)	46 (25%)	149 (25%)	235 (17%)	217 (29%)	
No surgery	232 (7.9%)	11 (6.0%)	25 (4.2%)	72 (5.2%)	124 (17%)	
Missing values	82	3	7	23	49	
**Radiotherapy**						**<0.001**
Yes	2,183 (75%)	153 (84%)	481 (80%)	1,134 (81%)	415 (56%)	
No	739 (25%)	30 (16%)	120 (20%)	263 (19%)	326 (44%)	
Missing values	81	3	7	22	49	
**Chemotherapy**						**<0.001**
Yes	1,231 (42%)	146 (80%)	370 (62%)	576 (41%)	139 (19%)	
No	1,691 (58%)	37 (20%)	231 (38%)	821 (59%)	602 (81%)	
Missing values	81	3	7	22	49	
**Hormonal therapy**						**0.004**
Yes	1,929 (66%)	101 (55%)	383 (64%)	947 (68%)	498 (67%)	
No	991 (34%)	82 (45%)	218 (36%)	450 (32%)	241 (33%)	
Missing values	83	3	7	22	51	
**Targeted therapy**						**<0.001**
Yes	378 (13%)	42 (23%)	102 (17%)	171 (12%)	63 (8.5%)	
No	2,544 (87%)	141 (77%)	499 (83%)	1,226 (88%)	678 (91%)	
Missing values	81	3	7	22	49	
**Status vital**						**<0.001**
Alive	2,482 (83%)	169 (91%)	572 (94%)	1,275 (90%)	466 (59%)	
Dead	521 (17%)	17 (9.1%)	36 (5.9%)	144 (10%)	324 (41%)	
**Mean follow-up duration (month)**	68 (51, 81)	68 (52, 80)	71 (60, 83)	70 (58, 83)	60 (28, 75)	
**Loss to follow-up (treated as censored)**	592 (20%)	37 (20%)	120 (20%)	297 (21%)	138 (17%)	

^1^ N (%); Median (IQR).

^2^ Pearson's Chi-squared test; Fisher's exact test.

Bold type indicates statistical significance.

NA, Not applicable.

**Table 2**; **Figure 2** show that the 5‐year OS rates varied by age. Patients aged 40–49 and 50–69 had the most favourable survival (95.3% and 92.3%, respectively), while those aged <40 and ≥70 years had lower survival (91.0% and 65.4%, respectively; P<0.001). For women aged 50–69 years, the screening target group, the mode of detection influenced outcomes. Screen‐detected cases achieved better survival (95.8% 5‐year OS) than interval‐detected and diagnosis‐detected cases. Supplementary analyses using refined age subgroups (**Supplementary Tables 4**, **5**; **Supplementary Figure 2**) showed that women aged 40–44 presented with slightly more aggressive tumour features than those aged 45–49 (poorly differentiated: 42.7% vs. 35.4%; SBR Grade III: 37.7% vs. 31.6%), though 5-year OS was comparable between the two subgroups (94.6% and 95.8%, respectively). Within the ≥70 group, 5-year OS differed markedly between women aged 70–74 (78.7%; 95% CI: 73.6–84.3%) and those ≥75 years (59.2%; 95% CI: 55.0–63.8%), highlighting the heterogeneity masked by the broad ≥70 category.

**Table 2:** 5-year overall survival rates stratified by age group.

Variable / Categories	All ages			<40			40–49			50–69			≥70		
	Events	5-year OS, %	p-value	Events	5-year OS,%	p-value	Events	5-year OS,%	p-value	Events	5-year OS,%	p-value	Events	5-year OS,%	p-value
**Overall survival**	389	85.8	NA	NA	NA	NA	NA	NA	NA	NA	NA	NA	NA	NA	NA
<40	15	91.0	**<0.001**	NA	NA	NA	NA	NA	NA	NA	NA	NA	NA	NA	NA
40–49	26	95.3		NA	NA		NA	NA		NA	NA		NA	NA	
50–69	99	92.3		NA	NA		NA	NA		NA	NA		NA	NA	
≥70	249	65.4		NA	NA		NA	NA		NA	NA		NA	NA	
Luxembourg	373	84.7	**<0.001**	13	90.4	0.473	24	94.9	0.529	94	91.8	0.231	242	64.3	0.034
Other country	16	94.8		2	94.3		2	97.7		5	95.9		7	85.8	
**Detection mode**															
Screen-detected	NA	NA	NA	NA	NA	NA	NA	NA	NA	23	95.8	**<0.001**	NA	NA	NA
Interval-detected	NA	NA	NA	NA	NA	NA	NA	NA	NA	11	94.3		NA	NA	NA
Diagnosis-detected	NA	NA	NA	NA	NA	NA	NA	NA	NA	64	87.9		NA	NA	NA
I	80	93.7	**<0.001**	0	100.0	**<0.001**	4	98.4	**<0.001**	20	96.9	**<0.001**	56	80.1	**<0.001**
II	127	85.8		9	88.2		8	95.9		27	92.6		83	67.5	
III	62	61.3		3	74.1		4	85.7		19	66.7		36	43.8	
IV	99	37.3		3	47.6		9	50.0		28	57.4		59	13.9	
Luminal A	59	91.1	**<0.001**	1	96.7	0.189	2	97.9	**0.002**	7	97.8	**<0.001**	49	73.7	**0.004**
Luminal B HER2-negative	95	86.4		3	91.2		6	96.2		29	90.8		57	70.0	
Luminal B HER2-positive	40	87.6		2	94.5		3	95.9		13	90.7		22	67.9	
HER2-positive (non-luminal)	11	88.3		0	100.0		2	91.1		2	95.6		7	65.0	
Triple-negative tumours	63	74.9		6	82.3		9	85.7		17	82.8		31	44.0	

NA, Not applicable; OS, Overall survival. Bold values indicate statistically significant results (p < 0.05).

**Figure 2:**
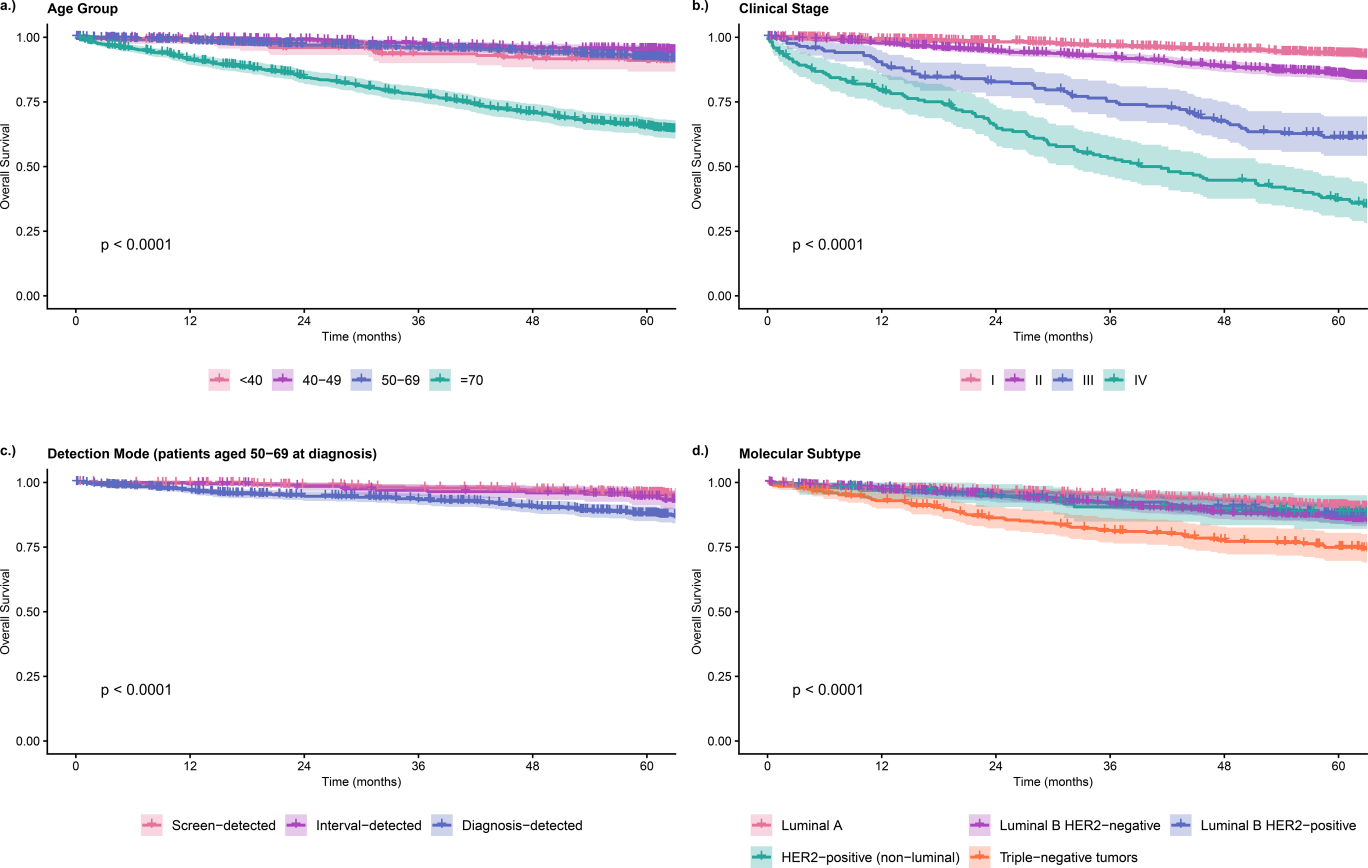
Kaplan-Meier plot comparing overall survival among **(A)** Age group (<40, 40–49, 50–59 and >70), **(B)** Clinical stage (I, II, III and IV) and **(C)** Detection mode (patients aged 50–69 at diagnosis: Screen-detected, Interval-detected, and Diagnosis-detected), and **(D)** Molecular subtypes (Luminal A, Luminal B HER2-negative, Luminal B HER2-positive, HER2-positive (non-luminal), and Triple-negative tumours).

The results of the univariate Cox proportional hazards model are shown in **Supplementary Table 2**. After adjusting for clinical stage, molecular subtype, and treatment factors (**Table 3**), advanced stages (III and IV) and triple-negative tumours emerged as unfavourable prognostic factors. In the overall model, stage IV disease had a hazard ratio (HR) of 2.92 (95% CI: 2.05–4.17; P<0.001), while triple‐negative tumours showed an HR of 2.39 (1.69, 3.38; P<0.001). Mastectomy (HR = 2.04; 95% CI: 1.54–2.70) and absence of surgery (HR = 8.58; 95% CI: 6.01–12.24) were associated with poorer survival in the overall model, reflecting disease complexity and tumour burden rather than causal treatment effects. Age-based analyses showed that older patients (≥70 years) had significantly worse outcomes, whereas in younger women (<40 years), triple‐negative status and absence of surgery were particularly unfavourable.

**Table 3:** Final multivariate Cox proportional hazards model by age group.

Variable / Categories	All ages		<40		40–49		50–69		≥70	
	HR (95% CI)	p-value	HR (95% CI)	p-value	HR (95% CI)	p-value	HR (95% CI)	p-value	HR (95% CI)	p-value
Country of residence *(Ref.: Luxembourg)*										
Other country	**0.57 (0.37, 0.87)**	**0.009**	0.26 (0.03, 2.10)	0.207	0.58 (0.18, 1.84)	0.357	0.50 (0.24, 1.06)	0.070	0.89 (0.47, 1.69)	0.719
Detection mode *(Ref.: Screen−detected)*										
Interval−detected	NA	NA	NA	NA	NA	NA	1.05 (0.51, 2.16)	0.888	NA	NA
Diagnosis−detected	NA	NA	NA	NA	NA	NA	**2.12 (1.20, 3.75)**	**0.009**	NA	NA
Clinical stage *(Ref.: I or II)*										
III	**2.42 (1.71, 3.44)**	**<0.001**	0.71 (0.08, 6.14)	0.755	2.63 (0.74, 9.31)	0.133	**3.32 (1.60, 6.91)**	**0.001**	**2.19 (1.40, 3.44)**	**0.001**
IV	**2.92 (2.05, 4.17)**	**<0.001**	4.44 (0.27, 72.30)	0.295	**32.00 (9.22, 111.00)**	**<0.001**	**3.54 (1.71, 7.32)**	**0.001**	**3.35 (2.15, 5.23)**	**<0.001**
Molecular subtypes *(Ref.: Luminal A)*										
Luminal B HER2−negative	1.22 (0.90, 1.65)	0.192	1.45 (0.15, 14.24)	0.752	0.76 (0.21, 2.76)	0.671	**2.99 (1.47, 6.09)**	**0.002**	1.09 (0.76, 1.58)	0.636
Luminal B HER2−positive	0.99 (0.68, 1.43)	0.947	1.02 (0.08, 13.46)	0.989	0.75 (0.15, 3.75)	0.729	**3.44 (1.57, 7.54)**	**0.002**	1.33 (0.82, 2.15)	0.252
HER2−positive (non−luminal)	0.59 (0.32, 1.08))	0.089	NE	0.998	1.93 (0.40, 9.41)	0.417	0.42 (0.05, 3.35)	0.412	0.73 (0.35, 1.52)	0.404
Triple−negative tumours	**2.39 (1.69, 3.38)**	**<0.001**	6.57 (0.74, 58.19)	0.091	**5.59 (1.62, 19.24)**	**0.006**	**5.53 (2.52, 12.12)**	**<0.001**	2.13 (1.31, 3.48)	**0.002**
Surgery *(Ref.: Breast−conserving surgery)*										
Mastectomy	**2.04 (1.54, 2.70)**	**<0.001**	**5.12 (1.42, 18.38)**	**0.012**	1.75 (0.69, 4.43)	0.235	0.99 (0.54, 1.81)	0.976	**1.68 (1.15, 2.44)**	**0.007**
No surgery	**8.58 (6.01, 12.24)**	**<0.001**	18.75 (1.31, 267.69)	0.031	0.86 (0.13, 5.85)	0.876	**3.64 (1.68, 7.88)**	**0.001**	**6.76 (4.45, 10.26)**	**<0.001**

Multivariate Cox regression, adjusted for all factors.

Bold type indicates statistical significance.

HR, Hazard ratio, CI, confidence interval, NA, Not applicable, NE, Not estimable (due to the zero frequency of events).

## Discussion

This study highlights the impact of age at diagnosis on BC prognosis, emphasising differences in tumour biology, treatment approaches, and survival outcomes. Previous research has established age as a key prognostic factor; however, results across studies remain heterogeneous due to differences in design, age groupings, and adjustment strategies (2, 3, 7, 8). The present study contributes to this debate by providing high-resolution data from a national cancer registry, reinforcing the need for age-adapted screening and treatment strategies.

In Luxembourg, the healthcare system operates on a compulsory social security model providing universal coverage through the CNS, covering most diagnostic and treatment services including surgery, chemotherapy, radiotherapy, and hormonal therapy (43). BC care follows national and European guidelines through public and private hospitals. The country’s high proportion of foreign-born residents and cross-border workers may influence access, continuity of care, and follow-up (44, 45).

Younger BC patients (<40 years) present with more aggressive disease characteristics, including higher prevalence of poorly differentiated and triple-negative tumours, requiring more aggressive and targeted therapeutic strategies. In our cohort, the 5-year OS for this group was 91.0%, which, although relatively high, was still lower than that observed in middle-aged (40–49 and 50–69) patients. These findings are consistent with prior studies demonstrating worse prognoses among young BC patients due to aggressive tumour biology and limited treatment response (15–17). EUSOMA recommendations emphasise that younger patients should receive specialised management approaches, including intensified systemic treatments, fertility preservation and psychosocial support, to improve long-term outcomes (13, 14, 46).

Patients aged 40–49 and 50–69 years achieved the most favourable survival (95.3% and 92.3%, respectively). Within the 50–69 screening target group, screen-detected cases in our cohort achieved the highest survival (95.8%). Although adjustment for tumour stage and treatment factors attenuated this benefit, the high proportion of early-stage diagnoses (stage I, 61%) among screen-detected cases (**Supplementary Table 3**) highlights the role of early detection in prognosis. These results underscore the value of organised screening in systematically reaching asymptomatic women, while also reflecting the efficiency of Luxembourg’s healthcare system in facilitating rapid diagnosis and timely treatment (12, 18, 26, 28).

Older patients (≥70 years) had poorer survival (5-year OS: 65.4%), a finding consistent with published evidence indicating the influence of advanced disease, higher comorbidity burden, and treatment de-escalation (47–49). The decline in chemotherapy use with age reflects both a higher prevalence of triple-negative and HER2-positive tumours in younger women and age-related treatment de-escalation independent of tumour biology. This pattern is evident across all molecular subtypes: among women with triple-negative tumours, chemotherapy receipt declined from 100% in those aged <40 to 50% in women aged 70–74 and 40% in those ≥75 years; a similar gradient was observed for Luminal B HER2-positive tumours (92.1% in <40 vs. 75% in 70–74 and 25.5% in ≥75), reinforcing that de-escalation occurs even in subtypes for which systemic treatment is standard of care (**Supplementary Table 6**). This is reflected in the marked decline in chemotherapy receipt from 41% in women aged 50–69 to 19% in those aged ≥70 (**Table 1**). Logistic regression adjusting for molecular subtype and clinical stage confirmed that chemotherapy use varies independently of tumour biology across all age subgroups. Compared with women aged 50–69, younger women were significantly more likely to receive chemotherapy (<40: OR = 5.41, 95% CI: 3.06–9.95; 40–44: OR = 2.27, 95% CI: 1.49–3.49; 45–49: OR = 2.13, 95% CI: 1.47–3.12; all p<0.001), while older women were significantly less likely to do so (70–74: OR = 0.45, 95% CI: 0.29–0.68; ≥75: OR = 0.05, 95% CI: 0.03–0.08; both p<0.001). As expected, molecular subtype was the strongest determinant of chemotherapy receipt (HER2-positive (non-luminal) tumours (OR = 108.09, 95% CI: 46.91–277.36), triple-negative tumours (OR = 28.54, 95% CI: 18.23–45.60), and Luminal B HER2-positive tumours (OR = 21.76, 95% CI: 14.62–32.87) were all strongly associated with chemotherapy use relative to Luminal A) confirming that the age-related decline occurs even after accounting for differences in tumour biology (**Supplementary Tables 6**, **7**). These findings reinforce the importance of geriatric oncology assessment over age-based decision-making, per European Society of Breast Cancer Specialists – International Society of Geriatric Oncology (EUSOMA-SIOG) guidelines (50). As Luxembourg has extended screening to 70–74 years, future evaluation should determine whether this policy improves early detection and outcomes in older women. Published evidence suggests benefits for selected healthy women, but the net value of screening in this age group remains uncertain (25). It should be noted that the 5-year OS of 65.4% observed in women ≥70 years in our cohort reflects overall mortality from all causes, not BC-specific mortality. Competing causes of death in this age group (including cardiovascular disease and other malignancies) substantially contribute to this estimate, and the true BC-attributable survival disadvantage in older women may be smaller than suggested by OS alone. Refined subgroup analyses further distinguish women aged 70–74 (5-year OS: 78.7%; 95% CI: 73.6–84.3%) from those ≥75 years (59.2%; 95% CI: 55.0–63.8%), a distinction with direct policy relevance given the 2024 PM expansion to ages 70–74. The relatively more favourable outcomes in the 70–74 group support the rationale for extending organised screening to this subgroup, while reinforcing the need for individualised assessment in women ≥75 years, as recommended by EUSOMA-SIOG guidelines (50). Within the 40–49 group, women aged 45–49, newly incorporated into the PM, showed tumour characteristics intermediate between those of women <40 and those aged 50–69, with 5-year OS comparable to that of women aged 40–44 (95.8% vs. 94.6%), providing a baseline against which the impact of expanded screening in this subgroup can be evaluated in future analyses. In Luxembourg, women aged 70–74 represent approximately 4% of the female population, whereas those ≥75 years account for 8% (51).

The associations of mastectomy and absence of surgery with worse OS in the multivariate model should not be interpreted causally. Surgery type serves as a surrogate for tumour burden, tumour-to-breast ratio, and overall clinical complexity, all of which are determinants of surgical indication. Mastectomy is typically selected for larger, multifocal, or centrally located tumours (42); absence of surgery reflects inoperable or very advanced disease, or cases where patient comorbidity or performance status precludes operative intervention (50). These factors are themselves strongly predictive of survival, and residual confounding by indication cannot be excluded even after multivariate adjustment for clinical stage and molecular subtype.

Although the population pyramid narrows in older age groups, the number of women who may benefit from early detection remains significant. Prior studies have shown that BC in older women is often associated with more favourable tumour biology (47, 50). These factors support the need for improved data to determine optimal screening cut-offs in older populations. The EUSOMA-SIOG guidelines suggest screening beyond 70 years should be individualised based on life expectancy, comorbidities, and treatment preferences (50). Our findings align with this recommendation, demonstrating that survival benefits are strongest within the organised screening cohort (ages 50–69), while older patients had poorer outcomes, necessitating further exploration of whether screening extensions would be beneficial for this population.

Several limitations should be considered when interpreting our findings. First, Luxembourg’s exceptionally high proportion of cross-border workers (47% of the workforce) and immigrants (47% of the resident population), introduces heterogeneity in healthcare access, screening participation, treatment continuity, and follow-up completeness (51, 52). Women receiving parts of their care abroad may not be fully captured in the RNC, and deaths occurring outside Luxembourg are not ascertained through national death certificate linkage, as follow-up for non-residents relies solely on hospital-based registry records. This likely introduces informative censoring, contributing to the 20% lost to follow-up observed in the cohort (44, 45). Sensitivity analyses confirmed that censored patients were disproportionately resident outside Luxembourg at diagnosis (38% vs. 8% among non-censored patients; **Supplementary Table 1**), consistent with cross-border residency as a driver of incomplete follow-up, as deaths occurring in neighbouring countries of residence are not captured through Luxembourg’s national death certificate linkage, rather than reflecting differential disease biology. Consequently, the observed survival advantage among non-Luxembourg residents (HR = 0.57) should be interpreted with caution, as it likely reflects ascertainment bias rather than a true biological or demographic effect. Future studies should address the impact of healthcare migration on BC outcomes and survival estimates. These characteristics limit direct comparability with EU-27 aggregate survival estimates, which typically reflect more homogeneous residential and follow-up patterns. Luxembourg’s observed survival rates may underestimate true BC-specific outcomes in the resident population, and this should be considered when contextualising our findings within a broader European framework. Second, survival was estimated with Kaplan–Meier overall survival rather than net survival or disease-specific survival. In older patients (≥70 years), this is particularly relevant, as non-cancer competing mortality substantially contributes to observed deaths, potentially overestimating the BC-attributable survival disadvantage in this group. While cause-of-death data are recorded in the RNC, cause-of-death ascertainment in older patients with comorbidities is subject to misclassification, limiting the reliability of disease-specific survival estimates. Net survival estimation using the Pohar–Perme estimator (53) would be preferable but requires accurate population-based life tables stratified by age and sex, which are challenging to construct in Luxembourg given its high migration turnover. Future studies should incorporate net survival methodology as these data improve. Third, lead-time and length-time biases may have inflated survival advantages in screen-detected cases (54). Fourth, the primary age categorisation (<40, 40–49, 50–69, ≥70) reflects clinical and policy relevance for the 2013–2018 study period but may mask differences within transitional subgroups. Supplementary analyses using refined subgroups (40–44, 45–49, 70–74, ≥75) are presented in **Supplementary Tables 4**, **5**; **Supplementary Figure 2**, but should be interpreted with caution given smaller sample sizes (40–44: N = 245; 70–74: N = 254). Fifth, the molecular classification used standard clinical markers, but emerging genomic classifiers could improve risk stratification and guide personalised treatment (46, 50). Sixth, SBR grade data were missing for 36% of patients (n=1,079), and differentiation grade for 20% (n=609). The high proportion of missing grading information likely reflects variability in pathological reporting practices across pathologists and over the study period. These gaps limit the granularity of tumour biology analyses and may introduce bias in grade-related subgroup comparisons. SBR grade was therefore excluded from all regression models; differentiation grade was retained in univariate analyses but not carried forward into the final multivariate model. Complete-case analysis was applied throughout. The extent of missingness should be considered when interpreting grade-related findings. Hormonal therapy allocation in clinical practice is based on direct ER/PR receptor status as reported in pathology records, independently of the research-derived molecular subtype classification used in this study. Consequently, patients with incomplete molecular subtype data may have received hormonal therapy on the basis of documented ER/PR positivity, which explains why the number of patients receiving hormonal therapy may exceed the count with a fully classified hormone receptor-positive subtype.

Despite these limitations, this present study provides the first population-based evidence in Luxembourg that age at diagnosis significantly influences BC prognosis. Younger women face aggressive disease and poorer outcomes, while older patients show lower OS, reflecting competing causes of mortality and comorbidity burden in addition to treatment de-escalation, rather than BC prognosis alone. Women aged 40–49 and 50–69 had the most favourable survival outcomes. Among women aged 50–69, the core screening target group during the study period, screen-detected cases achieved particularly high 5-year overall survival, underscoring the role of organised early detection in this group. These results provide a crucial baseline for evaluating the 2024 expansion of the PM to ages 45–74. Future analyses comparing cohorts before and after expansion will clarify whether these policy changes translate into improved detection and survival across a broader age spectrum.

## Data Availability

The data used in this study were obtained from a national cancer registry (RNC) and are subject to data use agreements that prohibit redistribution. Access to the dataset is restricted and granted only upon reasonable request and approval by the RNC Scientific Steering Committee. Requests to access the datasets should be directed to info@rnc.lu.
