# Supplementary material for: Age-related prognoses in a Luxembourgish breast cancer cohort
Source: Front Oncol. 2026 Jun 22;16:1763412. doi: 10.3389/fonc.2026.1763412 (PMC13333341; doi:10.3389/fonc.2026.1763412)
Supplement: Supplementary file 1 [file DataSheet1.docx]

**Supplementary Figure 1.** Eligibility criteria flowchart.

Excluded (n= 125)

♦  Sarcomas or Lymphomas (n= 20)

♦  Lacking follow-up data (n= 11)

♦  Second primary breast tumour (n= 94)

## Identification

## Analysis

## Inclusion

Assessed for eligibility

(n= 3,128 cancer cases / 3,034 women)

♦  C50 – Invasive breast cancer

♦  Diagnosed between 2013-2018

Analysed (n= 3,003 women)

Included (n= 3,003 women)

## Survival analysis

Analysed (n= 3,003 women)

)

## Cohort description

**Supplementary Figure 2 –** Overall survival by refined age subgroup.

**
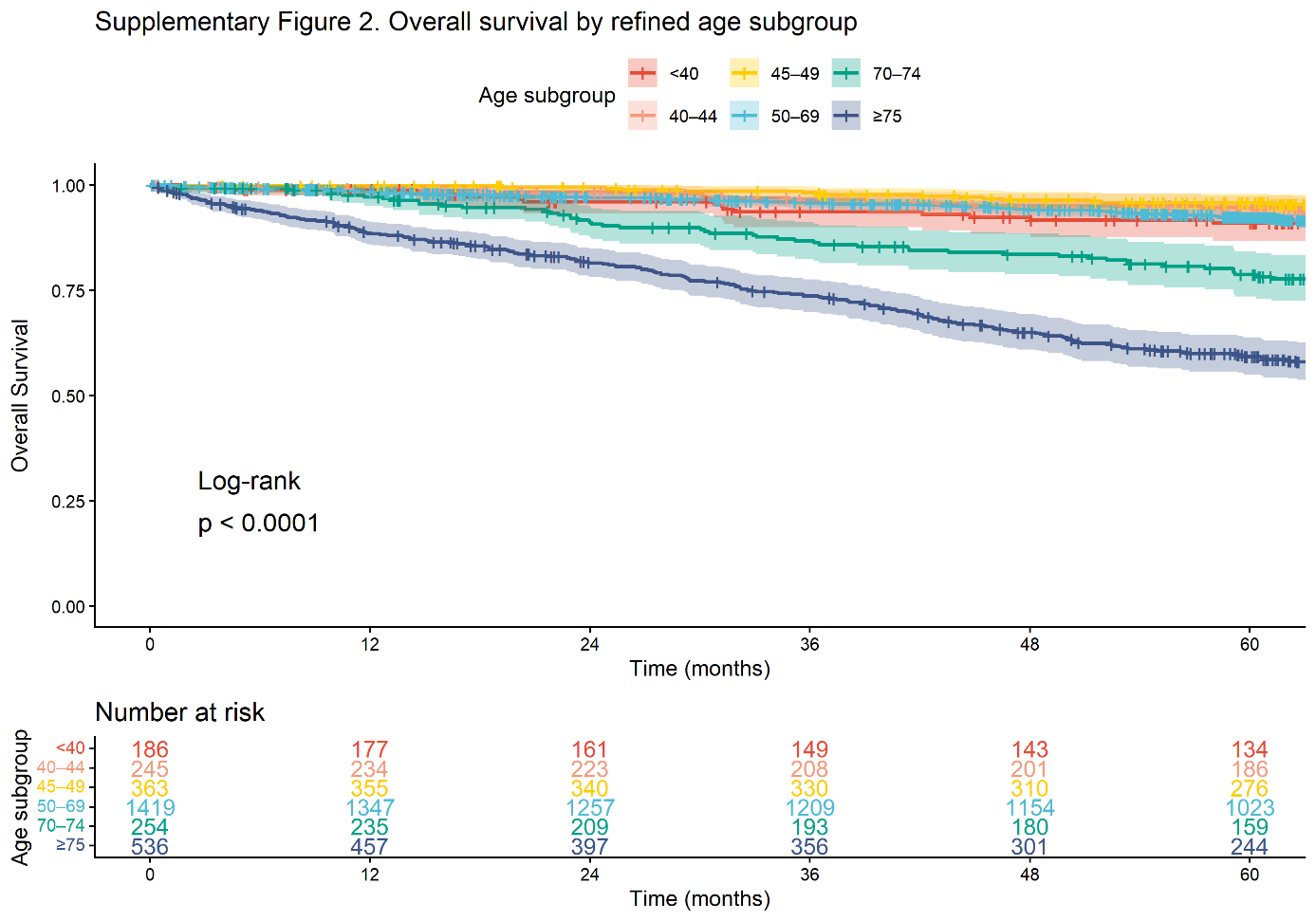
**
