# Supplementary material for: Age-related prognoses in a Luxembourgish breast cancer cohort
Source: Front Oncol. 2026 Jun 22;16:1763412. doi: 10.3389/fonc.2026.1763412 (PMC13333341; doi:10.3389/fonc.2026.1763412)
Supplement: Supplementary file 2 [file Table1.docx]

**Supplementary Table 1.** Comparison of censored and non-censored patients across key demographic and clinical characteristics.

| **Variable / Categories** | **Censored (Lost to Follow-Up)**  N = 592^1^ | **Non-Censored (Alive or Died)**  N = 2,411^1^ | **p-value**^2^ |
| --- | --- | --- | --- |
| **Age at Diagnosis (Years)** |  |  | 0.3 |
| <40 | 37 (6.3%) | 149 (6.2%) |  |
| 40–49 | 120 (20%) | 488 (20%) |  |
| 50–69 | 297 (50%) | 1,122 (47%) |  |
| ≥70 | 138 (23%) | 652 (27%) |  |
| **Country of Residence at Diagnosis** |  |  | <0.001 |
| Luxembourg | 365 (62%) | 2,219 (92%) |  |
| Other country | 227 (38%) | 192 (8.0%) |  |
| **Year of diagnosis** |  |  | <0.001 |
| 2013-14 | 120 (20%) | 864 (36%) |  |
| 2015-16 | 119 (20%) | 854 (35%) |  |
| 2017-18 | 353 (60%) | 693 (29%) |  |
| **Detection mode** |  |  | <0.001 |
| Screen-detected | 83 (26%) | 515 (43%) |  |
| Interval-detected | 31 (9.7%) | 183 (15%) |  |
| Diagnosis-detected | 204 (64%) | 500 (42%) |  |
| Missing values | 274 | 1,213 |  |
| **Histological diagnosis** |  |  | 0.2 |
| Ductal carcinoma | 326 (55%) | 1,384 (57%) |  |
| Lobular carcinoma | 223 (38%) | 896 (37%) |  |
| Others | 43 (7.3%) | 131 (5.4%) |  |
| **Differentiation grade** |  |  | 0.13 |
| Well/Moderately differentiated | 287 (70%) | 1,320 (66%) |  |
| Poorly/Undifferentiated differentiated | 121 (30%) | 666 (34%) |  |
| Missing values | 184 | 425 |  |
| **Clinical stage** |  |  | 0.008 |
| I | 299 (56%) | 1,116 (51%) |  |
| II | 188 (35%) | 791 (36%) |  |
| III | 28 (5.3%) | 144 (6.5%) |  |
| IV | 18 (3.4%) | 150 (6.8%) |  |
| Missing values | 59 | 210 |  |
| **Molecular subtypes** |  |  | <0.001 |
| Luminal A | 188 (37%) | 557 (32%) |  |
| Luminal B HER2-negative | 212 (42%) | 585 (34%) |  |
| Luminal B HER2-positive | 45 (8.9%) | 295 (17%) |  |
| HER2-positive (non-luminal) | 13 (2.6%) | 86 (4.9%) |  |
| Triple-negative tumours | 50 (9.8%) | 223 (13%) |  |
| Missing values | 84 | 665 |  |
| **Radiotherapy** |  |  | <0.001 |
| Yes | 467 (81%) | 1,716 (73%) |  |
| No | 107 (19%) | 632 (27%) |  |
| Missing values | 18 | 63 |  |
| **Chemotherapy** |  |  | 0.019 |
| Yes | 217 (38%) | 1,014 (43%) |  |
| No | 303 (62%) | 1,334 (57%) |  |
| Missing values | 18 | 63 |  |
| **Hormonal therapy** |  |  | <0.001 |
| Yes | 301 (53%) | 1,628 (69%) |  |
| No | 272 (47%) | 719 (31%) |  |
| Missing values | 19 | 64 |  |
| **Targeted therapy** |  |  | <0.001 |
| Yes | 49 (8.5%) | 329 (14%) |  |
| No | 525 (91%) | 2,019 (86%) |  |
| Missing values | 18 | 63 |  |

^1^ N (%)

^2^Pearson's Chi-squared test; Fisher's exact test
