# Supplementary material for: Age-related prognoses in a Luxembourgish breast cancer cohort
Source: Front Oncol. 2026 Jun 22;16:1763412. doi: 10.3389/fonc.2026.1763412 (PMC13333341; doi:10.3389/fonc.2026.1763412)
Supplement: Supplementary file 3 [file Table2.docx]

**Supplementary Table 2.** Univariate Cox proportional hazards model results.

| **Variable / Categories** | **All ages** | |  | **<40** | |  | **40‑49** | |  | **50‑69** | |  | | **≥70** | |  |
| --- | --- | --- | --- | --- | --- | --- | --- | --- | --- | --- | --- | --- | --- | --- | --- | --- |
|  | **HR (95% CI)** | **p-value** |  | **HR (95% CI)** | **p-value** |  | **HR (95% CI)** | **p-value** |  | **HR (95% CI)** | **p-value** | |  | **HR (95% CI)** | **p-value** | |
| **Age Group**  *(Ref.: 50‑69)* |  |  |  |  |  |  |  |  |  |  |  | |  |  |  | |
| <40 | 0.96 (0.58, 1.59) | 0.877 |  | NA | NA |  | NA | NA |  | NA | NA | |  | NA | NA | |
| 40‑49 | **0.57 (0.39, 0.82)** | **0.002** |  | NA | NA |  | NA | NA |  | NA | NA | |  | NA | NA | |
| ≥70 | **5.23 (4.29, 6.37)** | **<0.001** |  | NA | NA |  | NA | NA |  | NA | NA | |  | NA | NA | |
| **Country of Residence**  *(Ref.: Luxembourg)* |  |  |  |  |  |  |  |  |  |  |  | |  |  |  | |
| Other country | **0.42 (0.28, 0.63)** | **<0.001** |  | 0.59 (0.13, 2.57) | 0.479 |  | 0.72 (0.25, 2.03) | 0.532 |  | 0.66 (0.34, 1.31) | 0.235 | |  | **0.51 (0.27, 0.96)** | **0.038** | |
| **Year of Diagnosis**  *(Ref.: 2013‑14)* |  |  |  |  |  |  |  |  |  |  |  | |  |  |  | |
| 2015‑16 | **0.73 (0.60, 0.90)** | **0.003** |  | 1.42 (0.48, 4.18) | 0.524 |  | 0.95 (0.44, 2.04) | 0.892 |  | **0.54 (0.37, 0.80)** | **0.002** | |  | **0.75 (0.58, 0.97)** | **0.030** | |
| 2017‑18 | **0.76 (0.61, 0.95)** | **0.014** |  | 0.39 (0.08, 2.00) | 0.257 |  | 0.54 (0.21, 1.41) | 0.206 |  | **0.61 (0.40, 0.93)** | **0.023** | |  | 0.88 (0.67, 1.16) | 0.370 | |
| **Detection Mode**  *(Ref.:Screen‑detected)* |  |  |  |  |  |  |  |  |  |  |  | |  |  |  | |
| Interval‑detected | 1.44 (0.83, 2.50) | 0.197 |  | NA | NA |  | NA | NA |  | 1.33 (0.75, 2.36) | 0.333 | |  | NA | NA | |
| Diagnosis‑detected | **3.23 (2.23, 4.70)** | **<0.001** |  | NA | NA |  | NA | NA |  | **2.88 (1.96, 4.23)** | **<0.001** | |  | NA | NA | |
| **Histological Diagnosis**  *(Ref.: Ductal carcinoma)* |  |  |  |  |  |  |  |  |  |  |  | |  |  |  | |
| Lobular carcinoma | **0.71 (0.58, 0.86)** | **<0.001** |  | 0.34 (0.08, 1.49) | 0.152 |  | 0.94 (0.47, 1.87) | 0.851 |  | **0.58 (0.40, 0.84)** | **0.004** | |  | 0.82 (0.64, 1.05) | 0.123 | |
| Others | **2.02 (1.51, 2.70)** | **<0.001** |  | 1.58 (0.36, 7.02) | 0.546 |  | 1.62 (0.38, 6.95) | 0.515 |  | 1.57 (0.79, 3.12) | 0.195 | |  | **1.46 (1.04, 2.06)** | **0.030** | |
| **Differentiation Grade**  *(Ref.:Well/Moderately differentiated)* |  |  |  |  |  |  |  |  |  |  |  | |  |  |  | |
| Poorly/Undifferentiated | **1.59 (1.29, 1.95)** | **<0.001** |  | 1.03 (0.37, 2.91) | 0.950 |  | **2.74 (1.30, 5.79)** | **0.008** |  | **2.15 (1.49, 3.12)** | **<0.001** | |  | **1.79 (1.36, 2.36)** | **<0.001** | |
| **SBR**  (Ref.: Low Grade (I/II)) |  |  |  |  |  |  |  |  |  |  |  | |  |  |  | |
| High grade III | **1.60 (1.25, 2.04)** | **<0.001** |  | 0.66 (0.20, 2.17) | 0.496 |  | **2.94 (1.14, 7.60)** | **0.026** |  | **1.92 (1.23, 3.01)** | **0.004** | |  | **1.97 (1.42, 2.74)** | **<0.001** | |
| **Multifocality**  *(Ref.:Absence)* |  |  |  |  |  |  |  |  |  |  |  | |  |  |  | |
| Presence | **0.76 (0.63, 0.91)** | **0.004** |  | 1.53 (0.59, 3.96) | 0.382 |  | 0.98 (0.50, 1.92) | 0.947 |  | 1.16 (0.83, 1.62) | 0.393 | |  | **0.70 (0.54, 0.90)** | **0.006** | |
| **Laterality**  *(Ref.:Right)* |  |  |  |  |  |  |  |  |  |  |  | |  |  |  | |
| Left | 0.98 (0.82, 1.16) | 0.812 |  | 2.36 (0.83, 6.69) | 0.107 |  | 1.50 (0.76, 2.94) | 0.241 |  | 1.13 (0.82, 1.58) | 0.455 | |  | **0.77 (0.62, 0.96)** | **0.020** | |
| **Clinical T**  *(Ref.: T1)* |  |  |  |  |  |  |  |  |  |  |  | |  |  |  | |
| T2 | **2.47 (2.00, 3.06)** | **<0.001** |  | 3.17 (0.84, 11.95) | 0.089 |  | 2.03 (0.93, 4.46) | 0.077 |  | **2.51 (1.67, 3.78)** | **<0.001** | |  | **1.98 (1.51, 2.59)** | **<0.001** | |
| T3/T4 | **6.78 (5.37, 8.56)** | **<0.001** |  | **10.35 (2.45, 43.72)** | **0.001** |  | **8.45 (3.60, 19.84)** | **<0.001** |  | **8.36 (5.48, 12.76)** | **<0.001** | |  | **4.17 (3.08, 5.66)** | **<0.001** | |
| **Clinical N**  *(Ref.: N0)* |  |  |  |  |  |  |  |  |  |  |  | |  |  |  | |
| N+ (1, 2 and 3) | **2.82 (2.36, 3.38)** | **<0.001** |  | **9.11 (2.60, 31.98)** | **<0.001** |  | **3.67 (1.87, 7.20)** | **<0.001** |  | **4.17 (2.96, 5.86)** | **<0.001** | |  | **2.11 (1.67, 2.67)** | **<0.001** | |
| **Clinical M**  *(Ref.: M0)* |  |  |  |  |  |  |  |  |  |  |  | |  |  |  | |
| M1 | **7.77 (6.28, 9.61)** | **<0.001** |  | **10.22 (2.91, 35.89)** | **<0.001** |  | **16.30 (7.53, 35.26)** | **<0.001** |  | **10.39 (7.18, 15.04)** | **<0.001** | |  | **5.30 (3.96, 7.10)** | **<0.001** | |
| **Site of Metastasis**  *(Ref.: Non‑visceral)* |  |  |  |  |  |  |  |  |  |  |  | |  |  |  | |
| Visceral | 0.96 (0.61, 1.51) | 0.859 |  | NA | NA |  | 0.80 (0.17, 3.85) | 0.780 |  | 0.66 (0.25, 1.71) | 0.394 | |  | 0.84 (0.48, 1.48) | 0.552 | |
| **Clinical Stage**  *(Ref.: I)* |  |  |  |  |  |  |  |  |  |  |  | |  |  |  | |
| II | **2.30 (1.83, 2.89)** | **<0.001** |  | NE | NE |  | 1.80 (0.74, 4.37) | 0.191 |  | **2.28 (1.46, 3.59)** | **<0.001** | |  | **1.86 (1.40, 2.48)** | **<0.001** | |
| III | **6.64 (4.99, 8.84)** | **<0.001** |  | NE | NE |  | **6.53 (2.29, 18.58)** | **<0.001** |  | **9.74 (5.66, 16.75)** | **<0.001** | |  | **3.77 (2.62, 5.43)** | **<0.001** | |
| IV | **13.51 (10.43, 17.51)** | **<0.001** |  | NE | NE |  | **24.83 (9.66, 63.79)** | **<0.001** |  | **17.96 (11.35, 28.40)** | **<0.001** | |  | **8.47 (5.99, 11.98)** | **<0.001** | |
| **Molecular Subtypes**  *(Ref.: Luminal A)* |  |  |  |  |  |  |  |  |  |  |  | |  |  |  | |
| Luminal B HER2‑negative | **1.51 (1.14, 1.98)** | **0.003** |  | 4.05 (0.47, 34.67) | 0.997 |  | 1.19 (0.35, 4.07) | 0.779 |  | **4.94 (2.49, 9.82)** | **<0.001** | |  | 1.10 (0.79, 1.52) | 0.579 | |
| Luminal B HER2‑positive | **1.42 (1.02, 1.99)** | **0.041** |  | 1.45 (0.13, 15.97) | 0.202 |  | 1.33 (0.33, 5.39) | 0.689 |  | **5.20 (2.45, 11.05)** | **<0.001** | |  | 1.34 (0.87, 2.06) | 0.178 | |
| HER2‑positive (non‑luminal) | 1.17 (0.66, 2.05) | 0.597 |  | NE | NE |  | 3.70 (0.83, 16.56) | 0.087 |  | 1.65 (0.36, 7.53) | 0.518 | |  | 1.17 (0.58, 2.34) | 0.664 | |
| Triple‑negative tumours | **2.59 (1.89, 3.56)** | **<0.001** |  | 5.31 (0.64, 44.13) | 0.123 |  | **4.98 (1.57, 15.76)** | **0.006** |  | **8.46 (4.01, 17.87)** | **<0.001** | |  | **2.19 (1.44, 3.33)** | **<0.001** | |
| **BRCA1**  *(Ref.: Negative)* |  |  |  |  |  |  |  |  |  |  |  | |  |  |  | |
| Positive | 1.09 (0.44, 2.67) | 0.858 |  | 1.00 (0.10, 9.61) | 0.999 |  | 2.63 (0.76, 9.11) | 0.126 |  | 0.45 (0.06, 3.53) | 0.445 | |  | NA | NA | |
| **BRCA2**  *(Ref.: Negative)* |  |  |  |  |  |  |  |  |  |  |  | |  |  |  | |
| Positive | 0.96 (0.37, 2.52) | 0.938 |  | 0.84 (0.09, 8.20) | 0.879 |  | 1.59 (0.35, 7.25) | 0.550 |  | 1.11 (0.24, 5.24) | 0.893 | |  | NE | NE | |
| **Surgery**  *(Ref.: Breast‑conserving surgery)* |  |  |  |  |  |  |  |  |  |  |  | |  |  |  | |
| Mastectomy | **2.72 (2.19, 3.37)** | **<0.001** |  | 3.39 (1.14, 10.09) | 0.028 |  | **3.31 (1.60, 6.88)** | **0.001** |  | **2.07 (1.36, 3.15)** | **<0.001** | |  | **2.04 (1.53, 2.71)** | **<0.001** | |
| No surgery | **12.21 (9.76, 15.27)** | **<0.001** |  | **11.18 (2.77, 45.15)** | **<0.001** |  | **9.36 (3.03, 28.88)** | **<0.001** |  | **11.81 (7.80, 17.87)** | **<0.001** | |  | **6.34 (4.75, 8.47)** | **<0.001** | |
| **Radiotherapy**  *(Ref.: Yes)* |  |  |  |  |  |  |  |  |  |  |  | |  |  |  | |
| No | **3.55 (2.96, 4.26)** | **<0.001** |  | 0.74 (0.17, 3.27) | 0.696 |  | 1.71 (0.81, 3.57) | 0.157 |  | **2.34 (1.65, 3.33)** | **<0.001** | |  | **2.87 (2.25, 3.66)** | **<0.001** | |
| **Chemotherapy**  *(Ref.: Yes)* |  |  |  |  |  |  |  |  |  |  |  | |  |  |  | |
| No | **1.24 (1.03, 1.49)** | **0.024** |  | 0.28 (0.04, 2.12) | 0.218 |  | **0.40 (0.17, 0.93)** | **0.033** |  | **0.49 (0.35, 0.69)** | **<0.001** | |  | 0.87 (0.65, 1.16) | 0.340 | |
| **Hormonal Therapy**  *(Ref.: Yes)* |  |  |  |  |  |  |  |  |  |  |  | |  |  |  | |
| No | **1.61 (1.34, 1.93)** | **<0.001** |  | **3.51 (1.22, 10.13)** | **0.020** |  | **4.10 (1.99, 8.44)** | **<0.001** |  | **1.95 (1.39, 2.73)** | **<0.001** | |  | **1.41 (1.11, 1.81)** | **0.006** | |
| **Targeted Therapy**  *(Ref.: Yes)* |  |  |  |  |  |  |  |  |  |  |  | |  |  |  | |
| No | 1.18 (0.89, 1.56) | 0.242 |  | 1.02 (0.33, 3.17) | 0.972 |  | 0.93 (0.39, 2.26) | 0.881 |  | 0.93 (0.57, 1.51) | 0.776 | |  | 0.86 (0.58, 1.28) | 0.451 | |
| Bold type indicates statistical significance.  HR: Hazard ratio, CI: confidence interval, NA: Not applicable, NE: Not estimable (due to the zero frequency of events). | | | | | | | | | | | | | | | |  |
