# Supplementary material for: Age-related prognoses in a Luxembourgish breast cancer cohort
Source: Front Oncol. 2026 Jun 22;16:1763412. doi: 10.3389/fonc.2026.1763412 (PMC13333341; doi:10.3389/fonc.2026.1763412)
Supplement: Supplementary file 4 [file Table3.docx]

**Supplementary Table 3.** Tumour behaviour and clinical stage by detection mode.

| **Variable** | **Overall**,  N = 1,682^1^ | **Screen-detected**,  N = 696^1^ | **Interval-detected**,  N = 223^1^ | **Diagnosis-detected**,  N = 763^1^ | **p-value**^2^ |
| --- | --- | --- | --- | --- | --- |
| **Tumour behaviour** |  |  |  |  | <0.001 |
| In situ | 167 (9.9%) | 98 (14%) | 11 (4.9%) | 58 (7.6%) |  |
| Invasive | 1,515 (90%) | 598 (86%) | 212 (95%) | 705 (92%) |  |
| **Clinical stage** |  |  |  |  | <0.001 |
| 0 | 171 (11%) | 109 (17%) | 7 (3.4%) | 55 (7.9%) |  |
| I | 807 (52%) | 396 (61%) | 110 (54%) | 301 (43%) |  |
| II | 424 (27%) | 128 (20%) | 68 (33%) | 228 (33%) |  |
| III | 71 (4.6%) | 11 (1.7%) | 10 (4.9%) | 50 (7.2%) |  |
| IV | 74 (4.8%) | 8 (1.2%) | 8 (3.9%) | 58 (8.4%) |  |
| Missing values | 135 | 44 | 20 | 71 |  |
| ^1^n (%) | | | | | |
| ^2^Pearson's Chi-squared test | | | | | |
