# Supplementary material for: Age-related prognoses in a Luxembourgish breast cancer cohort
Source: Front Oncol. 2026 Jun 22;16:1763412. doi: 10.3389/fonc.2026.1763412 (PMC13333341; doi:10.3389/fonc.2026.1763412)
Supplement: Supplementary file 5 [file Table4.docx]

Supplementary Table 4. Demographic and clinical characteristics of BC cases stratified by refined age subgroup at diagnosis.

| **Characteristic** | **Overall**  N = 3,003^1^ | **<40**  N = 186^1^ | **40–44**  N = 245^1^ | **45–49**  N = 363^1^ | **50–69**  N = 1,419^1^ | **70–74**  N = 254^1^ | **≥75**  N = 536^1^ | **p-value**^2^ |
| --- | --- | --- | --- | --- | --- | --- | --- | --- |
| **Histological diagnosis** |  |  |  |  |  |  |  | **<0.001** |
| Ductal carcinoma | 1,710 (56.9%) | 122 (65.6%) | 149 (60.8%) | 193 (53.2%) | 801 (56.4%) | 131 (51.6%) | 314 (58.6%) |  |
| Lobular carcinoma | 1,119 (37.3%) | 51 (27.4%) | 83 (33.9%) | 157 (43.3%) | 567 (40.0%) | 97 (38.2%) | 164 (30.6%) |  |
| Others | 174 (5.8%) | 13 (7.0%) | 13 (5.3%) | 13 (3.6%) | 51 (3.6%) | 26 (10.2%) | 58 (10.8%) |  |
| **Differentiation grade** |  |  |  |  |  |  |  | **<0.001** |
| Well/Moderately differentiated | 1,607 (67.1%) | 61 (40.4%) | 114 (57.3%) | 199 (64.6%) | 822 (71.4%) | 137 (70.3%) | 274 (70.4%) |  |
| Poorly/Undifferentiated differentiated | 787 (32.9%) | 90 (59.6%) | 85 (42.7%) | 109 (35.4%) | 330 (28.6%) | 58 (29.7%) | 115 (29.6%) |  |
| Unknown | 609 | 35 | 46 | 55 | 267 | 59 | 147 |  |
| **SBR grade** |  |  |  |  |  |  |  | **<0.001** |
| Grade I | 347 (18.0%) | 7 (6.3%) | 27 (16.2%) | 43 (18.4%) | 191 (20.1%) | 30 (18.2%) | 49 (16.4%) |  |
| Grade II | 1,040 (54.1%) | 42 (37.8%) | 77 (46.1%) | 117 (50.0%) | 539 (56.8%) | 94 (57.0%) | 171 (57.4%) |  |
| Grade III | 537 (27.9%) | 62 (55.9%) | 63 (37.7%) | 74 (31.6%) | 219 (23.1%) | 41 (24.8%) | 78 (26.2%) |  |
| Unknown | 1,079 | 75 | 78 | 129 | 470 | 89 | 238 |  |
| **Multifocality** |  |  |  |  |  |  |  | **<0.001** |
| Presence | 1,046 (34.8%) | 79 (42.5%) | 113 (46.1%) | 134 (36.9%) | 497 (35.0%) | 71 (28.0%) | 152 (28.4%) |  |
| Absence | 1,957 (65.2%) | 107 (57.5%) | 132 (53.9%) | 229 (63.1%) | 922 (65.0%) | 183 (72.0%) | 384 (71.6%) |  |
| **Laterality** |  |  |  |  |  |  |  | 0.423 |
| Right | 1,495 (49.9%) | 87 (46.8%) | 134 (54.7%) | 169 (46.6%) | 716 (50.5%) | 126 (50.0%) | 263 (49.2%) |  |
| Left | 1,503 (50.1%) | 99 (53.2%) | 111 (45.3%) | 194 (53.4%) | 701 (49.5%) | 126 (50.0%) | 272 (50.8%) |  |
| Unknown | 5 | 0 | 0 | 0 | 2 | 2 | 1 |  |
| **Clinical T** |  |  |  |  |  |  |  | **<0.001** |
| T1 | 1,582 (58.4%) | 78 (47.6%) | 135 (61.1%) | 184 (57.0%) | 830 (64.8%) | 145 (64.4%) | 210 (42.3%) |  |
| T2 | 856 (31.6%) | 70 (42.7%) | 75 (33.9%) | 110 (34.1%) | 341 (26.6%) | 57 (25.3%) | 203 (40.9%) |  |
| T3 | 121 (4.5%) | 12 (7.3%) | 8 (3.6%) | 21 (6.5%) | 49 (3.8%) | 8 (3.6%) | 23 (4.6%) |  |
| T4 | 150 (5.5%) | 4 (2.4%) | 3 (1.4%) | 8 (2.5%) | 60 (4.7%) | 15 (6.7%) | 60 (12.1%) |  |
| Unknown | 294 | 22 | 24 | 40 | 139 | 29 | 40 |  |
| **Clinical N** |  |  |  |  |  |  |  | **<0.001** |
| N0 | 2,232 (77.2%) | 118 (66.3%) | 176 (74.3%) | 270 (75.4%) | 1,117 (81.5%) | 186 (76.9%) | 365 (72.1%) |  |
| N1 | 588 (20.3%) | 56 (31.5%) | 58 (24.5%) | 81 (22.6%) | 221 (16.1%) | 49 (20.2%) | 123 (24.3%) |  |
| N2 | 28 (1.0%) | 2 (1.1%) | 0 (0.0%) | 4 (1.1%) | 11 (0.8%) | 2 (0.8%) | 9 (1.8%) |  |
| N3 | 44 (1.5%) | 2 (1.1%) | 3 (1.3%) | 3 (0.8%) | 22 (1.6%) | 5 (2.1%) | 9 (1.8%) |  |
| Unknown | 111 | 8 | 8 | 5 | 48 | 12 | 30 |  |
| **Clinical M** |  |  |  |  |  |  |  | **<0.001** |
| M0 | 2,803 (94.3%) | 175 (96.2%) | 236 (96.7%) | 350 (97.2%) | 1,335 (95.1%) | 228 (91.6%) | 479 (90.0%) |  |
| M1 | 168 (5.7%) | 7 (3.8%) | 8 (3.3%) | 10 (2.8%) | 69 (4.9%) | 21 (8.4%) | 53 (10.0%) |  |
| Unknown | 32 | 4 | 1 | 3 | 15 | 5 | 4 |  |
| **Clinical stage** |  |  |  |  |  |  |  | **<0.001** |
| I | 1,415 (51.8%) | 63 (38.0%) | 119 (53.6%) | 161 (49.8%) | 757 (58.8%) | 123 (53.7%) | 192 (37.9%) |  |
| II | 979 (35.8%) | 84 (50.6%) | 90 (40.5%) | 128 (39.6%) | 398 (30.9%) | 73 (31.9%) | 206 (40.7%) |  |
| III | 172 (6.3%) | 12 (7.2%) | 5 (2.3%) | 24 (7.4%) | 64 (5.0%) | 12 (5.2%) | 55 (10.9%) |  |
| IV | 168 (6.1%) | 7 (4.2%) | 8 (3.6%) | 10 (3.1%) | 69 (5.4%) | 21 (9.2%) | 53 (10.5%) |  |
| Unknown | 269 | 20 | 23 | 40 | 131 | 25 | 30 |  |
| **Molecular subtypes** |  |  |  |  |  |  |  | **<0.001** |
| Luminal A | 745 (33.1%) | 30 (19.7%) | 42 (21.9%) | 74 (27.4%) | 393 (36.9%) | 66 (35.5%) | 140 (35.9%) |  |
| Luminal B HER2-negative | 797 (35.4%) | 41 (27.0%) | 76 (39.6%) | 93 (34.4%) | 368 (34.6%) | 81 (43.5%) | 138 (35.4%) |  |
| Luminal B HER2-positive | 340 (15.1%) | 38 (25.0%) | 37 (19.3%) | 46 (17.0%) | 148 (13.9%) | 20 (10.8%) | 51 (13.1%) |  |
| HER2-positive (non-luminal) | 99 (4.4%) | 7 (4.6%) | 9 (4.7%) | 16 (5.9%) | 47 (4.4%) | 3 (1.6%) | 17 (4.4%) |  |
| Triple-negative tumors | 273 (12.1%) | 36 (23.7%) | 28 (14.6%) | 41 (15.2%) | 108 (10.2%) | 16 (8.6%) | 44 (11.3%) |  |
| Unknown | 749 | 34 | 53 | 93 | 355 | 68 | 146 |  |
| **Surgery** |  |  |  |  |  |  |  | **<0.001** |
| Breast-conserving surgery | 2,042 (69.9%) | 126 (68.9%) | 166 (68.6%) | 261 (72.7%) | 1,089 (78.0%) | 171 (67.9%) | 229 (46.8%) |  |
| Mastectomy | 647 (22.1%) | 46 (25.1%) | 67 (27.7%) | 82 (22.8%) | 235 (16.8%) | 58 (23.0%) | 159 (32.5%) |  |
| No surgery | 232 (7.9%) | 11 (6.0%) | 9 (3.7%) | 16 (4.5%) | 72 (5.2%) | 23 (9.1%) | 101 (20.7%) |  |
| Unknown | 82 | 3 | 3 | 4 | 23 | 2 | 47 |  |
| **Radiotherapy** |  |  |  |  |  |  |  | **<0.001** |
| Yes | 2,183 (74.7%) | 153 (83.6%) | 191 (78.9%) | 290 (80.8%) | 1,134 (81.2%) | 181 (71.8%) | 234 (47.9%) |  |
| No | 739 (25.3%) | 30 (16.4%) | 51 (21.1%) | 69 (19.2%) | 263 (18.8%) | 71 (28.2%) | 255 (52.1%) |  |
| Unknown | 81 | 3 | 3 | 4 | 22 | 2 | 47 |  |
| **Chemotherapy** |  |  |  |  |  |  |  |  |
| Yes | 1,231 (42.1%) | 146 (79.8%) | 154 (63.6%) | 216 (60.2%) | 576 (41.2%) | 81 (32.1%) | 58 (11.9%) | **<0.001** |
| No | 1,691 (57.9%) | 37 (20.2%) | 88 (36.4%) | 143 (39.8%) | 821 (58.8%) | 171 (67.3%) | 431 (88.1%) |  |
| Unknown | 81 | 3 | 3 | 4 | 22 | 2 | 47 |  |
| **Hormonal therapy** |  |  |  |  |  |  |  | **0.014** |
| Yes | 1,929 (66.1%) | 101 (55.2%) | 150 (62.0%) | 233 (64.9%) | 947 (67.8%) | 172 (68.3%) | 326 (66.9%) |  |
| No | 991 (33.9%) | 82 (44.8%) | 92 (38.0%) | 126 (35.1%) | 450 (32.2%) | 80 (31.7%) | 161 (33.1%) |  |
| Unknown | 83 | 3 | 3 | 4 | 22 | 2 | 49 |  |
| **Targeted therapy** |  |  |  |  |  |  |  | **<0.001** |
| Yes | 378 (12.9%) | 42 (23.0%) | 43 (17.8%) | 59 (16.4%) | 171 (12.2%) | 25 (9.9%) | 38 (7.8%) |  |
| No | 2,544 (87.1%) | 141 (77.0%) | 199 (82.2%) | 300 (83.6%) | 1,226 (87.8%) | 227 (90.1%) | 451 (92.2%) |  |
| Unknown | 81 | 3 | 3 | 4 | 22 | 2 | 47 |  |
| **Vital status** |  |  |  |  |  |  |  | **<0.001** |
| Alive | 2,482 (82.7%) | 169 (90.9%) | 232 (94.7%) | 340 (93.7%) | 1,275 (89.9%) | 196 (77.2%) | 270 (50.4%) |  |
| Dead | 521 (17.3%) | 17 (9.1%) | 13 (5.3%) | 23 (6.3%) | 144 (10.1%) | 58 (22.8%) | 266 (49.6%) |  |
| **Mean follow-up duration (months)** | 68 (51, 81) | 68 (52, 80) | 72 (60, 80) | 71 (60, 85) | 70 (58, 84) | 66 (37, 78) | 56 (23, 73) | **<0.001** |
| ^1^n (%); Median (IQR). P-values from Pearson's chi-squared test or Kruskal-Wallis test. | | | | | | | | |
| ^2^Pearson's Chi-squared test; Kruskal-Wallis rank sum test | | | | | | | | |
