# Supplementary material for: Age-related prognoses in a Luxembourgish breast cancer cohort
Source: Front Oncol. 2026 Jun 22;16:1763412. doi: 10.3389/fonc.2026.1763412 (PMC13333341; doi:10.3389/fonc.2026.1763412)
Supplement: Supplementary file 6 [file Table5.docx]

Supplementary Table 5. Five-year overall survival rates stratified by refined age subgroup.

| **Age subgroup** | **N** | **Events (deaths)** | **At risk at 60 months** | **5-year OS (%)** | **95% CI lower (%)** | **95% CI upper (%)** |
| --- | --- | --- | --- | --- | --- | --- |
| <40 | 186 | 15 | 134 | 91.0 | 86.8 | 95.5 |
| 40–44 | 245 | 12 | 186 | 94.6 | 91.7 | 97.6 |
| 45–49 | 363 | 14 | 276 | 95.8 | 93.7 | 98.0 |
| 50–69 | 1,419 | 99 | 1,023 | 92.3 | 90.8 | 93.7 |
| 70–74 | 254 | 48 | 159 | 78.7 | 73.6 | 84.3 |
| ≥75 | 536 | 201 | 244 | 59.2 | 55.0 | 63.8 |
| OS: Overall survival; CI: Confidence interval. Log-rank test p-value = >0.001. | | | | | | |
