# Supplementary material for: Age-related prognoses in a Luxembourgish breast cancer cohort
Source: Front Oncol. 2026 Jun 22;16:1763412. doi: 10.3389/fonc.2026.1763412 (PMC13333341; doi:10.3389/fonc.2026.1763412)
Supplement: Supplementary file 7 [file Table6.docx]

Supplementary Table 6. Chemotherapy use by molecular subtype and age subgroup.

| **Group**^1^ | **Molecular subtype**^1^ | **No chemotherapy**^1^ | **Chemotherapy**^1^ | **p-value**^1^ |
| --- | --- | --- | --- | --- |
|  |  |  |  |  |
| <40 | Luminal A | 15 (51.7%) | 14 (48.3%) | <0.001 |
|  | Luminal B HER2-negative | 7 (17.5%) | 33 (82.5%) |  |
|  | Luminal B HER2-positive | 3 (7.9%) | 35 (92.1%) |  |
|  | HER2-positive (non-luminal) | 0 (0%) | 7 (100%) |  |
|  | Triple-negative tumors | 0 (0%) | 36 (100%) |  |
|  |  |  |  |  |
| 40–44 | Luminal A | 31 (73.8%) | 11 (26.2%) | <0.001 |
|  | Luminal B HER2-negative | 24 (32%) | 51 (68%) |  |
|  | Luminal B HER2-positive | 7 (18.9%) | 30 (81.1%) |  |
|  | HER2-positive (non-luminal) | 0 (0%) | 9 (100%) |  |
|  | Triple-negative tumors | 1 (3.8%) | 25 (96.2%) |  |
|  |  |  |  |  |
| 45–49 | Luminal A | 53 (72.6%) | 20 (27.4%) | <0.001 |
|  | Luminal B HER2-negative | 34 (37%) | 58 (63%) |  |
|  | Luminal B HER2-positive | 4 (8.7%) | 42 (91.3%) |  |
|  | HER2-positive (non-luminal) | 2 (12.5%) | 14 (87.5%) |  |
|  | Triple-negative tumors | 2 (5%) | 38 (95%) |  |
|  |  |  |  |  |
| 50–69 | Luminal A | 341 (87.2%) | 50 (12.8%) | <0.001 |
|  | Luminal B HER2-negative | 186 (51.1%) | 178 (48.9%) |  |
|  | Luminal B HER2-positive | 32 (21.8%) | 115 (78.2%) |  |
|  | HER2-positive (non-luminal) | 0 (0%) | 47 (100%) |  |
|  | Triple-negative tumors | 18 (17.5%) | 85 (82.5%) |  |
|  |  |  |  |  |
| 70–74 | Luminal A | 61 (92.4%) | 5 (7.6%) | <0.001 |
|  | Luminal B HER2-negative | 47 (58.8%) | 33 (41.2%) |  |
|  | Luminal B HER2-positive | 5 (25%) | 15 (75%) |  |
|  | HER2-positive (non-luminal) | 2 (66.7%) | 1 (33.3%) |  |
|  | Triple-negative tumors | 8 (50%) | 8 (50%) |  |
|  |  |  |  |  |
| ≥75 | Luminal A | 127 (96.9%) | 4 (3.1%) | <0.001 |
|  | Luminal B HER2-negative | 118 (94.4%) | 7 (5.6%) |  |
|  | Luminal B HER2-positive | 35 (74.5%) | 12 (25.5%) |  |
|  | HER2-positive (non-luminal) | 6 (35.3%) | 11 (64.7%) |  |
|  | Triple-negative tumors | 24 (60%) | 16 (40%) |  |
| ^1^n (%); p-value from Pearson's chi-squared test. | | | | |
