# Supplementary material for: Age-related prognoses in a Luxembourgish breast cancer cohort
Source: Front Oncol. 2026 Jun 22;16:1763412. doi: 10.3389/fonc.2026.1763412 (PMC13333341; doi:10.3389/fonc.2026.1763412)
Supplement: Supplementary file 8 [file Table7.docx]

Supplementary Table 7. Adjusted odds ratios for chemotherapy use (logistic regression: chemotherapy ~ age subgroup + molecular subtype + clinical stage).

| **Variable** | **OR** | **95% CI lower** | **95% CI upper** | **p-value** |
| --- | --- | --- | --- | --- |
| Age <40 | 5.41 | 3.06 | 9.95 | <0.001 |
| Age 40–44 | 2.27 | 1.49 | 3.49 | <0.001 |
| Age 45–49 | 2.13 | 1.47 | 3.12 | <0.001 |
| Age 70–74 | 0.45 | 0.29 | 0.68 | <0.001 |
| Age ≥75 | 0.05 | 0.03 | 0.08 | <0.001 |
| Luminal B HER2-negative | 4.84 | 3.62 | 6.53 | <0.001 |
| Luminal B HER2-positive | 21.76 | 14.62 | 32.87 | <0.001 |
| HER2-positive (non-luminal) | 108.09 | 46.91 | 277.36 | <0.001 |
| Triple-negative tumors | 28.54 | 18.23 | 45.60 | <0.001 |
| Stage II | 4.61 | 3.55 | 6.02 | <0.001 |
| Stage III | 10.74 | 6.27 | 18.89 | <0.001 |
| Stage IV | 3.84 | 2.31 | 6.44 | <0.001 |
| OR: Odds ratio; CI: Confidence interval. Reference categories: age 50-69 years, Luminal A subtype, Stage I. Complete cases: n = 2035. Excluded due to missing data on subtype or stage: n = 968. | | | | |
