# Supplementary material for: Age-related prognoses in a Luxembourgish breast cancer cohort
Source: Front Oncol. 2026 Jun 22;16:1763412. doi: 10.3389/fonc.2026.1763412 (PMC13333341; doi:10.3389/fonc.2026.1763412)
Supplement: Supplementary file 9 [file Table8.docx]

## Supplementary Table 8. Chemotherapy intent by age group at diagnosis.

| **Variable / Categories** | **All ages**  **(N=3,003)** | **<40**  **(N=186)** | **40-49**  **(N=608)** | **50-69**  **(N=1,419)** | **>=70**  **(N=790)** | **p-value** |
| --- | --- | --- | --- | --- | --- | --- |
| No | 1,691 (56.3%) | 37 (19.9%) | 231 (38%) | 821 (57.9%) | 602 (76.2%) | <0.001 |
| Yes - Neoadjuvant | 258 (8.6%) | 42 (22.6%) | 91 (15%) | 102 (7.2%) | 23 (2.9%) |  |
| Yes - Adjuvant | 822 (27.4%) | 84 (45.2%) | 243 (40%) | 407 (28.7%) | 88 (11.1%) |  |
| Yes - Neo + Adjuvant | 47 (1.6%) | 8 (4.3%) | 11 (1.8%) | 25 (1.8%) | 3 (0.4%) |  |
| Yes - Chemo without surgery | 60 (2%) | 5 (2.7%) | 9 (1.5%) | 28 (2%) | 18 (2.3%) |  |
| *Missing values* | *125* | *10* | *23* | *36* | *56* |  |
| *N (%); p-value from Pearson's Chi-squared test (excluding Missing values).*  *Treatment intent is not explicitly recorded in the registry. Chemotherapy intent was approximated from registry treatment records by comparing the first recorded chemotherapy date with the first recorded surgery date.*  ***Neoadjuvant****: chemotherapy administered prior to surgery only;*  ***Adjuvant:*** *chemotherapy after surgery only;*  ***Neo + Adjuvant:*** *chemotherapy episodes recorded both before and after surgery;*  ***Chemo without surgery:*** *consistent with metastatic or inoperable disease.*  *Missing values include patients with no chemotherapy information (n=81) and patients who received chemotherapy but had insufficient date information to classify intent (n=44).* | | | | | | |
